# Comparison of Health and Risk Factors of Older, Working-age Australians, Italians and Italian-born Migrants to Australia, with Data from an Italian (PASSI), and an Australian (SAMSS) Risk Factor Surveillance System

**DOI:** 10.1007/s10903-017-0654-9

**Published:** 2017-09-26

**Authors:** A. W. Taylor, E. Dal Grande, P. Fateh-Moghadam, A. Montgomerie, L. Battisti, H. Barrie, C. Kourbelis, S. Campostrini

**Affiliations:** 1 Population Research & Outcome Studies, Discipline of Medicine, The University of Adelaide, Adelaide, Australia; 2 Department of Health, Health Observatory, Trento, Italy; 3 Hugo Centre for Population and Migration, The University of Adelaide, Adelaide, Australia; 4 Department of Economics, Ca’ Foscari University, Venice, Italy

**Keywords:** Italy, Australia, Risk factors, Surveillance, Migration

## Abstract

Italian-born migrants (post-WWII) are the largest non-English-speaking background migrant group in South Australia. A cross-sectional, inter-country comparison using independent samples (40–69 years of age) from two (one in Australia, one in Italy) similar risk factor and chronic disease surveillance systems. None of the three groups (Italians, Australian-born and Italian-born Australians) had definitively worse health although the Italians had high rates for four of the seven risk factors reported (current high blood pressure, current high cholesterol, current smoking, eating less than five fruit and/or vegetables per day) than Australian-born and Italian-born Australians. Italian-born Australians had higher rates for insufficient physical activity, overweight/obese, poor self-reported health and diabetes. Australian respondents were more likely to report having two or more drinks of alcohol per day. Issues facing an ageing population require appropriate health care needs and an assessment of structural or cultural barriers to health services.

## Background

Australia is one of the most multicultural and ethnically diverse countries in the world [1, 2] with over a quarter of Australians born overseas [3]. Immigration from Italy has been an important component of Australia’s migration history with around 250,000 immigrating to Australia between 1947 and 1998 [4]. Until recently, Italians comprise the largest non-English speaking (NES) group in Australia [5]. While this has decreased in Australia in recent years, with an increase in Asian and Indian migration, Italians in South Australia (SA) continue to be the biggest NES migrant group [3].

Health selectivity associated with pre-immigration screening has been an important aspect of Australian’s immigration policy [6]. The health requirements and eligibility criteria has resulted in immigrants on arrival in Australia reporting better than average health and this has been reflected in longer life expectancy, lower death rates, lower hospitalisation rates and lower rates of risk factors [7, 8]. It is also argued that migrants self-select with pre-dominantly healthy individuals prepared to undertake the onerous task and stress often associated with most forms of migration [6, 8]. However, illness rates and risk factors has increased as duration of stay increases with this ‘healthy migrant effect’ declining as migrants adopt the risk factor behaviours [6, 9–11].

The main contemporary demographic issues associated with Italian immigration to Australia are the ageing of the Italian-born Australians with large number of second and subsequent generations [12]. Migrant health studies often overlook the relatively large proportion of current working age, Italian-born Australians. This study aims to analyse data on 40–69 years olds from two risk factor surveillance systems: Progessi Delle Aziende Sanitane per la Salute in Italia (PASSI) and the South Australian Monitoring and Surveillance System (SAMSS) to provide up-to-date information to policy makers to deliver appropriate, equitable and culturally sensitive services for Italian migrants. The analysis will compare the working age migrants from Italy to SA with both origin and host populations. As argued by Anson [13] it is important to compare with origin populations, as that is where formative years are often spent, acknowledging that early-life economic, social, cultural and political aspects of life affect health outcomes.

## Methodology

In this cross-sectional, inter-country comparison using independent samples, the data from two relatively similar risk factor and chronic disease surveillance systems have been utilised.

SAMSS is an ongoing telephone surveillance system designed to regularly monitor chronic disease, risk factors and other health-related issues [14]. Commencing in July 2002, a cross-sectional sample is randomly selected each month from all households in SA with a telephone number listed in the Electronic White Pages (EWP). A letter of introduction is sent to the selected household and the person who was last to have a birthday is chosen for interview.

Trained interviewers, via a Computer Assisted Telephone Interview (CATI) system, conducts the interviews in English. Each interview takes approximately 15–20 min. At least ten call-backs are made to the telephone number selected. Replacement interviews for persons who could not be contacted or interviewed are not permitted. A minimum of 600 randomly selected people (of all ages) are interviewed each month. The current analysis used data collected from July 2002 to December 2015 for respondents aged 40–69 years. The response rates of SAMSS for this period ranged between 51.4 and 75.2% (median = 64.9). All respondents gave informed consent to undertaking the interview. Ethics approval was obtained from the ethics committee of the Department of Health and Ageing, SA (HREC Number 14/SAH/200). Other details on SAMSS are available elsewhere [15].

PASSI was established in 2007 and is based on data collected via Local Health Units (LHU) each month. Primary approach letters are sent to each selected person. Data are collected in each of the 21 Italian regions via telephone calls from trained LHU staff and a random sample of 18 to 69 years adults are interviewed each month. At least six call-backs are made before selecting a substitute person of the same sex and age group. A national centre coordinates data management. Response rates are high (80+%). The interview takes approximately 20 min. Ethics approval was obtained from the Ethical Commission of the Istituto Superiore di Sanita (The Italian National Institute for Health). Other details on PASSI methodology are available elsewhere [16–18].

For both datasets body mass index (BMI) was derived from self-reported weight and height and recoded into three categories (underweight/normal, overweight and obese) [19]. Respondents were asked the number of serves of fruit and vegetables consumed each day with SAMSS being two separate questions and PASSI combining the questions into one. These questions were recoded into inadequate fruit and vegetable consumption (<5 vegetables and/or fruits per day) [16, 20].

Regarding physical activity, the SA respondents were asked the time spent undertaking walking, moderate or vigorous physical activity over the past week. To determine if respondent’s level of physical activity is sufficient to provide a health benefit, sufficient physical activity was defined has having 150 min or more of total time spent with vigorous activity multiplied by a factor of two to account for its greater intensity. The respondents were then classified as undertaking no activity, activity but not sufficient and sufficient activity [21]. PASSI participants were asked on how many days and for how long they undertook moderate and vigorous activity. Respondents with no activity, or activity but not sufficient activity, were classified as insufficient physical activity in order to allow PASSI data to be compared to the SA data.

SA included two alcohol questions recommended by the [Australian] National Health and Medical Research Council [22]. These are ‘how often do you drink alcohol’ and ‘on a day when you drink alcohol how many drinks do you usually have’. PASSI respondents were asked ‘during the past 30 days how many days did you have at least one drink of any alcohol beverage’ and ‘how many drinks did you drink on average’ [16]. Respondents who drank two or more standard drinks on any day were classified into a risky category.

PASSI respondents who reported smoking at least 100 cigarettes in their lifetime and who smoked either every day or some days were defined as current smokers. Respondents who reported smoking at least 100 cigarettes in their lifetime and not smoked in the previous 6 months were ex-smokers. Non-smokers were those never smoked 100 cigarettes. For SAMSS, smoking status (current, ex or non) was assessed by a single question. The proportion of current smokers for both data-sets was assessed.

All respondents were asked if they had ever been told by a doctor they had diabetes. They were also asked if a doctor told them they had high blood pressure (HBP) or high cholesterol and/or were on medication for the condition. Respondents were considered as having at least one risk factor if they had at least one of the following; current HBP, current high cholesterol, insufficient physical activity, overweight/obese, current smoker, risky alcohol intake, or insufficient fruit and vegetable consumption.

SA respondents were asked to rate their health as poor, fair, good, very good or excellent. PASSI respondents rated their current health status as excellent, good, fair, poor or very poor. A self-reported health status variable was created comparing SA poor overall health status with very poor for PASSI.

Comparable demographic and socioeconomic variables included age, gender, work status, highest education attainment, marital status, and the number of days in the previous 4 weeks (30 days) the respondent had been unable to work or carry out normal duties because of health.

Both samples were weighted to correct for disproportionality of the sample with respect to the population of interest and to allow for some of the bias to be compensated as a result of the non-contacted households or for any gender or age-group discrepancies. The SAMSS data were weighted each month by age, sex and area of residence to reflect the structure of the population in SA to the latest Australian Bureau of Statistics (ABS) Census data, and probability of selection in the household. Approximately 60–70% of the total SA households are included in the EWP. PASSI data were weighted by gender and age at the regional level based on annual Italian National Institute of Statistics figures to compensate for different sampling proportion in the LHU (covering over 90% of the residents in Italy). Eligible respondents are randomly selected from the LHU registries, and as no citizen can access any health service without being registered, the registries are highly reliable and regularly updated [16].

### Data Analysis

Data were analyzed using SPSS for Windows Version 20.0 and Stata Version 9.2. The analyses for SAMSS was limited to 40 to 69 years of age as PASSI upper age limit is 69 years. The lower age was determined because of the relatively skewed older Italian-born Australians owing to the decline in migration from Italy to Australia in recent decades compared to early post-war decades. For PASSI 2007 data was excluded since the PASSI system not being national until 2008. The SAMSS data included data from July 2002 and was limited to respondents born in Australia, and the PASSI data to those born in Italy. Data were age-adjusted to the World Health Organization (WHO) world standard population by a direct standardization method.

## Results

In total data from 27,604 Australian, 547 Italian-born Australians and 178,575 Italian respondents were used. The mean age was 52.6 years (SD 8.31, range 40–69) for Australian-born participants, 58.0 years (SD 7.26, range 40–69) for Italian-born Australian participants and 53.5 (SD 8.62, range 40–69) for Italian participants. Table 1 highlights the demographic profile of the study participants with Italian-born Australians more likely to be older and married and less likely to have a high level of education or to be employed. The risk factor and health profile of the respondents are detailed in Table 2 in crude and age standardised format. Italian respondents had higher standardised prevalence rates of current HBP, current high cholesterol, current smoking, eating less than five fruit and/or vegetables per day and more combined risk factors than Australian-born and Italian-born Australians. Italian-born Australians had higher rates for insufficient physical activity, overweight/obese, poor self-reported health and diabetes. Australian respondents were more likely to report having two or more drinks of alcohol per day.

**Table 1:** Demographic profile of Australian-born, Italian-born Australians and Italians—aged 40–69 years, SAMSS July 2002–December 2015 and PASSI January 2008–December 2015

	Australian (SAMSS)		Italian-born Australians (SAMSS)		Italian (PASSI)	
	n	% (95% CI)	n	% (95% CI)	n	% (95% CI)
Sex (% male)	13,742	49.8 (49.2–50.4)	270	49.4 (45.2–53.5)	87,759	49.4 (49.3–49.5)
Age						
40–44 years	6208	22.5 (22.0–23.0)	39	7.1 (5.2–9.5)	33,757	19.2 (19.0–19.4)
45–49 years	4969	18.0 (17.6–18.5)	39	7.2 (5.3–9.6)	34,579	20.0 (19.8–20.2)
50–54 years	5490	19.9 (19.4–20.4)	94	17.1 (14.2–20.5)	29,309	16.1 (15.9–16.3)
55–59 years	4157	15.1 (14.6–15.5)	123	22.4 (19.1–26.1)	27,595	15.3 (15.1–15.5)
60–64 years	3943	14.3 (13.9–14.7)	136	24.9 (21.5–28.7)	27,985	15.4 (15.2–15.6)
65–69 years	2837	10.3 (9.9–10.6)	117	21.4 (18.2–25.0)	25,350	14.0 (13.8–14.2)
Work status^a^ (% employed)	19,644	71.2 (70.6–71.7)	268	48.9 (44.7–53.1)	107,882	59.9 (59.6–60.2)
Education^a^						
No schooling to completed primary school	506	1.8 (1.7–2.0)	136	25.0 (21.5–28.8)	22,433	12.3 (12.1–12.4)
Some high school/completed high school/TAFE or trade certificate or diploma	20,942	76.0 (75.5–76.5)	360	65.9 (61.9–69.8)	131,276	74.9 (74.6–75.1)
Bachelor degree or higher	6120	22.2 (21.7–22.7)	50	9.1 (7.0–11.8)	20,272	12.9 (12.7–13.1)
Marital status^a^						
Married or living with partner	22,063	80.0 (79.6–80.5)	459	83.8 (80.5–86.7)	136,740	77.3 (77.0–77.5)
Separated or divorced	2714	9.8 (9.5–10.2)	47	8.5 (6.4–11.1)	13,123	7.0 (6.9–7.2)
Widowed	656	2.4 (2.2–2.6)	25	4.6 (3.1–6.7)	7229	3.9 (3.8–4.0)
Never married	2134	7.7 (7.4–8.1)	17	3.1 (2.0–4.9)	21,407	11.8 (11.6–12.0)
Economic indicator						
Unable to work or carry out normal duties because of health	4283	15.5 (15.1–16.0)	95	17.4 (14.5–20.8)	29,255	17.7 (17.5–18.0)
Total	27,604	100.0	547	100.0	178,575	100.0

The weighting of the data can result in rounding discrepancies or totals no adding

*CI* confidence interval

^a^Not stated not included

**Table 2:** Prevalence of risk factors among Australian-born, Italian-born and Italian-born Australians (40–69 years), SAMSS July 2002–December 2015 and PASSI January 2008–December 2015

	Australian			Italian-born Australians			Italian		
		Crude	Age standardised^b^		Crude	Age standardised^b^		Crude	Age standardised^b^
	n	%	% (95% CI)	n	%	% (95% CI)	n	%	% (95% CI)
Current high blood pressure	5852	22.5	22.3 (21.8–22.8)	154	30.3	23.4 (19.2–27.6)	49,455	29.5	27.6 (27.4–27.9)
Current high cholesterol	4959	19.1	18.9 (18.4–19.3)	133	26.2	20.6 (16.5–24.6)	49,318	30.4	29.0 (28.7–29.3)
Insufficient physical activity	12,983	50.8	50.8 (50.2–51.4)	303	60.6	57.2 (51.7–62.7)	96,510	56.3	56.3 (55.9–56.6)
Overweight/obese	17,687	67.5	67.4 (66.8–68.0)	385	74.1	71.6 (66.5–76.7)	89,749	51.1	50.1 (49.8–50.5)
Current smoker	4420	16.0	16.1 (15.7–16.5)	84	15.3	20.8 (16.1–25.6)	43,592	25.3	26.0 (25.7–26.2)
<5 serves veg/fruit per day	16,490	59.8	59.9 (59.3–60.4)	303	55.5	54.6 (49.2–60.0)	157,252	88.6	88.8 (88.3–89.3)
Alcohol—2 + standard drinks/day	5520	32.1	31.2 (30.7–31.8)	42	14.1	16.0 (11.7–20.3)	38,018	19.9	19.7 (19.5–19.9)
At least one health risk factor^a^	26,059	94.4	94.4 (94.1–94.7)	523	95.5	94.8 (92.3–97.4)	175,779	98.5	98.5 (98.0–99.0)
At least two health risk factors^a^	21,404	77.5	77.5 (77–78)	431	78.8	77.0 (72.4–81.7)	155,624	87.5	87.0 (86.5–87.4)
Poor self-reported health	1142	4.1	4.1 (3.9–4.4)	46	8.3	7.0 (4.8–9.2)	8663	5.2	4.9 (4.8–5.0)
Diabetes	2176	7.9	7.9 (7.6–8.2)	81	14.8	12.0 (9.3–14.8)	12,124	7.2	6.4 (6.3–6.5)

^a^Respondents were considered as having at least one risk factor if they had at least one of the following; current high blood pressure, current high cholesterol, insufficient physical activity, overweight/obese, current smoker, drank more than two standard alcoholic drinks on any day or insufficient fruit and vegetable consumption

^b^Age-adjusted to the World Health Organization (WHO) world standard population by direct standardization

## Discussion

This research has compared the demographic, health status and risk factor profile of older (40–69 years old) Italian migrants to SA with those of the same age range living in SA who were Australian-born, and those of the same age range born and living in Italy. Studies have shown the existence and diluting of the healthy migrant effect with migrants having better health on arrival but moving towards the host country as years since migration passes [6, 23]. Our data, although not having year of arrival, shows that the Italian migrants are between origin and host country for three of the risk factors (HBP, high cholesterol, current smoking and at least one risk factor) and are worse in four of the nine health-related variables assessed (insufficient physical activity, obesity, overall health status and diabetes). The two risk factors that the Italian migrants had not ‘overtaken’ the Australian-born rates were inadequate fruit and vegetable consumption and risky alcohol consumption.

Understanding and interpreting the results of this study is complex and hampered by the many additional factors and dimensions that are associated with cross-country comparisons and migration per se. Not adequately covered in this analysis are the selection criteria associated with migration, birthplace, and reason for migration. Additional important weaknesses include the lack of data on the number of years since migration and the age of immigration although data from the ABS suggests that Italian migrants to South Australia in the 40–69 years age group have a mean time in Australia of 47.9 years (SD 9.08, range 1–63 years) [24]. Risk factors differ by regions within countries is acknowledged, by gender and by a host of different socio-economic and socio-demographic variables and this analysis is somewhat of a ‘broad brush’ approach only. Some risk factors in Italy vary largely across regions and a future analysis that included the region from which the Italian-born Australians migrated could further enhance the results. In addition, SAMSS interviews were undertaken in English only with the understanding that English proficiency of this age group of Italian immigrants is high, and some wording differences in some of the questions that could result in bias. The analyses are also further limited by the cut-off of 69 years determined by the PASSI criteria. Inclusion of older persons may have provided different results. Notwithstanding these weaknesses, the strengths of this study are the use of large population based samples that used similar questions and similar interviewing technique. Often migrant studies in Australia combine all European populations under one category and the benefits of SAMSS was the ability to assess a broad range of details specifically on Italian migrants acknowledging that the country of origin pays an important role in health differences [25, 26].

Smoking rates of Italian-born Australians has previously been shown to be higher than Australian-born especially for males [27–29]. Overall smoking rates are declining in most developed countries [30] including Australia and Italy [31, 32]. This difference in rates could be attributed to the more stringent smoking policies enforced in Australia where successful policy endeavours and considerable public health effort has been expended in targeting smoking although smoking bans in Italy are also (since 2005) widespread [18]. The Italian-born Australians are perhaps influenced by the high tax rates and considerable longer public places smoking bans although the nearly 5% point higher prevalence in Italian-born Australians is an obvious target for intervention. The high Italian smoking rate in this age group is also a cause for concern and a target for Italian public health professionals.

In terms of HBP, the Italian-born Australian’s standardised prevalence rate was between the birth and the host countries. An early study (1977) of CVD risk factors compared Italian-born (20–79 years olds) matched by age, sex, place of residence to Australian-born and results indicated that Italian-born men had lower blood pressure although this was not the case for women [28]. This same study also reported lower levels of high cholesterol. A further Australian study, using data from 1980 to 1989, also reported similar results with the Italian migrants having lower HBP and high cholesterol rates [27]. If the healthy migrant effect was operating in these earlier studies the obvious benefit has now been removed with the age standardised rates higher for HBP and high cholesterol for the Italian migrants compared to the Australian-born. In both of these previous studies systolic blood pressure and various cholesterol levels were measured and reported rather than the self-report methodology of our study.

BMI has been increasing in both South Australia [33] and Italy over recent decades [32], although rates in Italy were below average when 26 industrialised countries were compared [34]. Previous Australian research in the 1980s and 1990s reported Italian migrants having higher BMI than their Australian-born counterparts [27, 28] and our results indicate this trend has continued. Previous studies indicated that the Italians were shorter and heavier than the Australian-born. Our data shows that the Italian-born migrants mean height is 6.1 cms shorter although the mean weight is 3.8 kgs heavier than the Australian-born comparison group (data not shown). Notwithstanding, the overweight/obese prevalence estimates for both Italian-born Australians and Australian-born are alarmingly high especially when compared to the Italian figures.

Previous research has shown that both Italian and Greek immigrants to Australia aged 40–69 years are more likely to have increased type 2 diabetes with the suggestion that although BMI may play a part, other risk factors are also of importance [7, 29]. Our results indicate that the diabetes prevalence rate for Italian-born Australians is much higher than the other two birth categories. This has previously been reported [6, 7, 29]. Cunningham et al. [23] call for research such as ours comparing host and origin countries to understand the role of genetics and environmental concerns. The wide variation between the two Italian-born groups indicates that environmental and cultural issues seem to play an important role.

The one risk factor that Italian-born migrants were better than both the host and origin countries is fruit and vegetable consumption with origin country having high rates of inadequate fruit and vegetables. A previous Italy study has shown that fruit and vegetable consumption is below the recommended range across all age groups [32], although perception of what constitutes a serve of vegetables (eg pizza toppings, vegetable sauces for pastas) is sometimes believed to not be counted as a serve of vegetables. Studies have assessed current Italian adherence to the Mediterranean diet [35]—a diet which includes high fruit and vegetable consumption. While younger age groups tend to be moving to a more westernised diet, adults of the age group we are studying are still more likely to maintain a Mediterranean type diet [35, 36].

An early study (1977) determined that the Italian-born men consumed more alcohol than the Australian-born but the opposite was true for women [28]. This was also reported in the later 1980s analyses [27]. Our study reports Australian-born participants being more likely to report higher alcohol consumption than both the comparison groups. While Australian alcohol consumption rates are going down [37], overall rates are still relatively high and especially among baby boomer populations [38].

## Conclusion

This study has been undertaken in an endeavour to provide health planners and policy makers information on current demographic, socio-economic and health status profile of this priority population, so that culturally appropriate health services to Italian-born immigrants, that takes into account their heritage, can be provided. If one asks the questions–are the Italian-born migrants to Australia in better health than their Australian born counterparts, as proposed by Anikeeva et al. [7], our data indicates that the answer would be no with the exception in adequate fruit and vegetable consumption, and lower alcohol consumption. If we ask the question, are Italian migrants in better health than their Italian counterparts, our data indicates the answer is confused with positives for HBP, high cholesterol, smoking, and fruit and vegetables consumption but not for the other variables assessed (physical activity, BMI, alcohol, quality of life and diabetes). The limitations associated with the data collection and analyses of this study are important considerations to take into account when assessing these findings.

The issues affecting migrant health especially for this age group are important considerations. Compared to United States and United Kingdom where approximately 13% of the population are foreign-born [23, 29], Australia’s 27.7% and SA’s 23.7% demand serious consideration [3]. The duality of Italians being SA biggest NES background migrant group, coupled with issues facing an ageing population, requires appropriate health care needs and an assessment of structural or cultural barriers to health services. A range of culturally and linguistic appropriate programs and policies have been implemented in Australia aimed at assisting migrants access appropriate health services and age successfully. With the healthy migrant effect a distant fallacy, continuation of these programs and policies are warranted more than ever.

## Abbreviations

ABS: Australian Bureau of Statistics
BMI: Body mass index
CATI: Computer assisted telephone interviewing
CVD: Cardio vascular disease
EWP: Electronic white pages
HBP: High blood pressure
LHU: Local health unit
NES: Non English speaking
PASSI: Progessi Delle Aziende Sanitane per la Salute in in Italia
SAMSS: South Australian monitoring and surveillance System
SD: Standard deviation
WHO: World Health Organization
